# Medium-Term Weight Loss and Remission of Comorbidities Following Endoscopic Sleeve Gastroplasty: a Systematic Review and Meta-analysis

**DOI:** 10.1007/s11695-023-06778-x

**Published:** 2023-09-13

**Authors:** Matyas Fehervari, Michael G Fadel, Laith Omar Khalaf Alghazawi, Bibek Das, María Rita Rodríguez-Luna, Silvana Perretta, Andrew Wan, Hutan Ashrafian

**Affiliations:** 1 Department of Surgery and Cancer, Imperial College London, London, UK; 2 Department of Upper Gastrointestinal and Bariatric Surgery, St. George’s University Hospitals NHS Foundation Trust, London, UK; 3 IHU-Strasbourg, Institute of Image-Guided Surgery, Strasbourg, France; 4 Research Institute against Digestive Cancer (IRCAD), Strasbourg, France; 5 ICube Laboratory, Photonics Instrumentation for Health, Strasbourg, France; 6 Department of Digestive and Endocrine Surgery, University of Strasbourg, Strasbourg, France

**Keywords:** Obesity, Endoscopic sleeve gastroplasty, Weight loss, Comorbidities

## Abstract

This systematic review and meta-analysis aimed to determine the short- and medium-term weight loss outcomes and comorbidity resolution following endoscopic sleeve gastroplasty. Our search identified 35 relevant studies containing data from 7525 patients. Overall, pooled short-term (12 months) total weight loss (TWL) was 16.2% (95% CI 13.1–19.4%) in 23 studies (*n* = 5659). Pooled medium-term TWL was 15.4% (95% CI 13.7–17.2%) in 10 studies (*n* = 4040). Diabetes resolution was 55.4% (95% CI 46–64%), hypertension resolution was 62.8% (95% CI 43–82%), dyslipidaemia resolution was 56.3% (95% CI 49–63%), and obstructive sleep apnoea resolution was 51.7% (95% CI 16.2–87.3%) in four studies (*n* = 480). This pooled analysis demonstrates that ESG can induce durable weight loss and resolution of obesity-associated comorbidities in patients with moderate obesity.

**Graphical Abstract:**

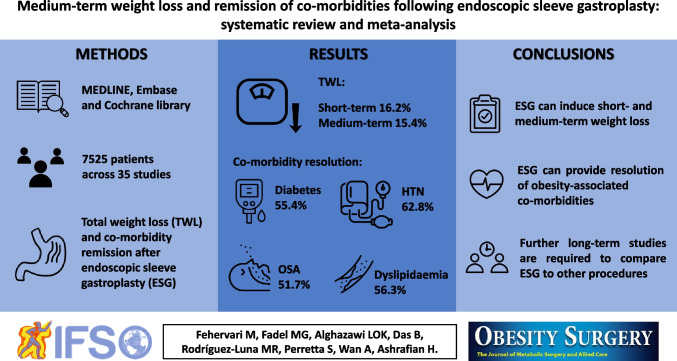

## Introduction

The management of obesity has significantly evolved over the last decade. The first effective surgical treatment of morbid obesity and associated conditions was Roux-en-Y gastric bypass. This was followed by laparoscopic sleeve gastrectomy (LSG), which rapidly became the most performed bariatric operation globally [1–3]. These surgical procedures are associated with significant short- and long-term weight loss as well as remission of obesity-related comorbidities [4, 5].

More recently, less invasive procedures such as endoscopic sleeve gastroplasty (ESG) have been introduced [6]. This procedure aims to create a sleeve-like stomach by intraluminal suturing but keeps the fundus and antrum intact. The reduction of the gastric volume leads to delayed gastric emptying and early satiety. The procedure generates similar gut hormonal changes to conventional bariatric surgery [7, 8]. This fully endoscopic procedure has several appealing advantages compared to laparoscopic operations, including a truly scarless technique, shorter hospital stay and improved perioperative outcomes [9–11]. Endoscopic bariatric procedures could be an alternative solution to bariatric surgery in elderly or surgical unfit patients [12]. Given the increasing utilisation of ESG, it is important to understand the impact on clinically relevant outcomes. Thus, we performed a systematic review and meta-analysis of reported weight loss outcomes and rates of remission of obesity-related comorbidities following ESG over a 5-year period.

## Methods

### Search Strategy

A systematic literature search was performed using the following electronic databases: Medline, Embase and the Cochrane library between January 1995 and December 2022. The full search strategy has been provided as supplementary material (Supp. 1). The following MeSH terms along with their synonyms were used in all possible combinations: ‘endoscopic sleeve gastroplasty’, ‘endoscopic gastric sleeve’, ‘non-surgical sleeve’, ‘non-surgical gastric sleeve’, ‘morbidity’, ‘quality of life’, ‘diabetes mellitus’, ‘hypertension’, 'dyslipidaemia' and ‘obstructive sleep apnoea’. Studies identified from the search strategy were entered into Covidence (Victoria, Australia) for bibliographic management and duplicates removal. Two authors (MF and MGF) independently identified relevant studies, and any discrepancies were resolved by consensus with the help of a third author (HA).

This systematic literature search and meta-analysis was conducted based on a prospectively developed protocol and is reported according to the Preferred Reporting Items for Systematic Reviews and Meta-Analyses (PRISMA) statement [13]. The review was registered on PROSPERO Centre for Reviews and Dissemination in December 2022 (registration number: CRD42022387320).

### Inclusion and Exclusion Criteria

The following criteria were applied for inclusion in the study:

i) Randomised controlled trials (RCT), prospective or retrospective cohort studies, case (control) studies, cross-sectional studies
ii) Patients who had undergone ESG for obesity
iii) Reported outcomes of interest: weight loss and resolution of comorbidities
iv) Original full-text articles in the English language

Animal studies, reviews, abstracts, conference presentations, case reports, editorials and unpublished studies were excluded from the analysis.

### Data Extraction and Quality Assessment

A standardised data extraction form was developed on Covidence, and three authors (MF, MGF and LA) independently extracted all relevant data: study design, sample size, patient gender and age, comorbidities, mean body mass index (BMI), mean percentage of excess weight loss (%EWL) and mean percentage of total weight loss (%TWL) at 12 months (short-term) and 2–5 years (medium-term) and resolution of obesity-related comorbidities.

The longest available follow-up data was collected from each study unless there was a significant loss to follow-up reported. The majority of the studies reported outcomes according to Brethauer et al.; however, not all studies described how weight loss, comorbidities and remission outcomes were reported [14]. As the majority of studies were non-randomised, the risk of bias was assessed using the Newcastle-Ottawa Scale [15]. Any discrepancy was resolved by group discussion. The definition of medium-term was based on Mahawar 2018, and all articles were included in this category if they had at least 18 months of follow-up data [16]*.* The strength of clinical data and subsequent recommendations were graded according to Ho et al. and Bellomo et al. [17, 18].

### Statistical Analysis

Data were analysed using Stata Software (Version 15.1. StataCorp LCC, TX). Pooled weighted mean differences, and standardised mean differences were analysed by random effects meta-analysis. All studies with relevant data were included in the analysis. Statistical heterogeneity was calculated using the *I*^2^ statistic. This was graded as low (*I*^2^ < 30%), moderate (*I*^2^ = 30–60%) or high (*I*^2^ > 60%) based on the Cochrane Handbook for Systematic Reviews of Intervention.

## Results

### Study Selection

**Fig. 1:**
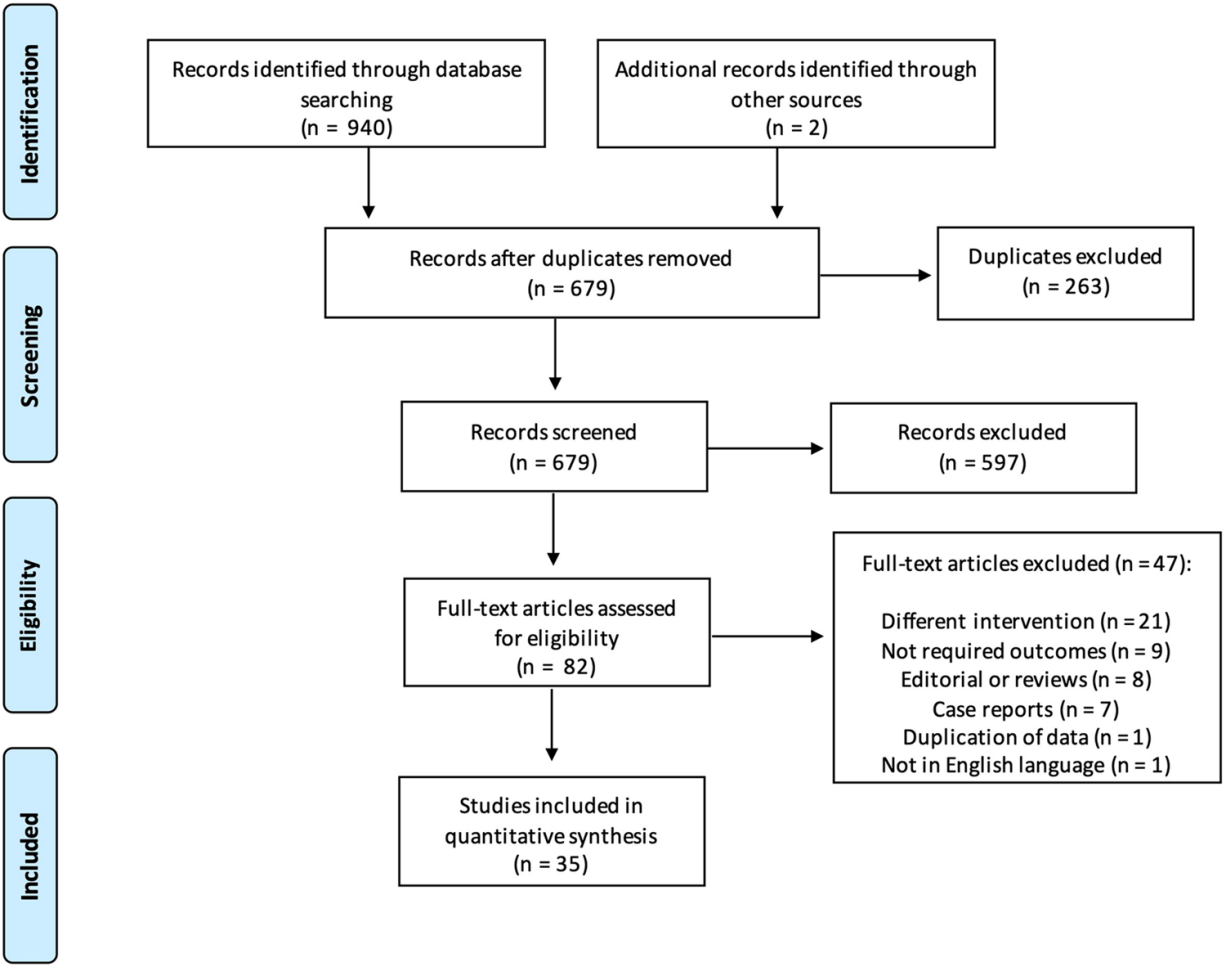
Flow diagram: process for selection of studies. Adapted from PRISMA 2009 flow diagram [13]

The search identified 940 relevant citations. After removing duplicate results, 679 articles were screened for titles and abstracts, and 82 studies were included in the full-text review. A total of 46 articles were excluded; thus, 32 non-randomised and 3 randomised studies were eligible for inclusion in the meta-analysis [6, 8, 12, 19–50]. The process of study selection is reported in Fig. 1.

### Characteristics of Studies

The included studies contained data from 7525 patients (mean age 42.2 years, 6461 (85.9%) female patients, mean BMI = 37.7 kg/m^2^). Twenty-eight of the studies were considered high quality, and four studies had high risk of bias according to the Newcastle-Ottawa scale (Supp 2.). The mean follow-up time following ESG was 17.4 months (range 6–60 months). The exact characteristics of the included studies are presented in Table 1.

**Table 1:** Summary of published series of endoscopic sleeve gastroplasty. *BMI* body mass index, *ESG* endoscopic sleeve gastroplasty, *GORD* gastro-oesophageal reflux disease, *HTN* hypertension, *LSG* laparoscopic sleeve gastrectomy, *OSA* obstructive sleep apnoea, *SD* standard deviation

Study	Year	Sample size (n)	% Male (n)	Mean age ± (SD), years	Mean Weight ± (SD), kg	Mean BMI ± (SD), kg/m^2^	Mean HbA1c ± (SD), %	Incidence of diabetes (n)	Incidence of HTN (n)	Incidence of OSA (n)	Incidence of other comorbidities (n)	Mean follow-up period, months	Study design and grade of study
Lopez-Nava et al. [36]	2017	248	27% (67)	44.5 ± 10		37.8 ± 5.6						24	Retrospective cohort Level III
Abu Dayyeh et al. [8]	2017	25	16% (4)	47.6 ± 10		35.5 ± 2.6						20	Prospective cohort Level III
Sharaiha et al. [38]	2017	91	32% (29)	43.86 ± 11.26		38.6 ± 7	6.1 ± 1.1	18	31		Abnormal LFTs 65	24	Prospective cohort Level III
Saumoy et al. [39]	2018	128	33% (42)	43.62 ± 11.37		38.92 ± 6.95						12	Prospective cohort Level III
Morales et al. [29]	2018	148	18% (27)	41.53 ± 10	98.7 ± 17	35.11 ± 5.5						18	Retrospective cohort Level III
Sartoretto et al. [41]	2018	112	31% (35)	45.1 ± 11.7		37.9 ± 6.7		14	26	16	GORD 34	6	Retrospective cohort Level III
Kumar et al. [42]	2018	77	36% (28)	41.3 ± 1.1	99.4 ± 1.8	36.1 ± 0.6						12	Prospective cohort Level III
Novikov et al. [27]	2018	91	23% (21)	42 ± 12		38.6 ± 6.9	5.8 ± 0.98	20	18	15	Dyslipidaemia 13	12	Retrospective cohort Level III
Alqahtani et al. [22]	2019	1000	10% (103)	34.4 ± 9.5		33.3 ± 4.5		17	17		Dyslipidaemia 32	18	Prospective cohort Level III
Fayad et al. [23]	2019	108	52% (23)	48		43.07		ESG 2	ESG 15	LSG 1	GORD 22	6	Retrospective cohort Level III
Barrichello et al. [40]	2019	193	23% (45)	42.3 ± 9.6	93.4 ± 10.31	34.11 ± 2.97						12	Retrospective cohort Level III
Cheskin et al. [43]	2019	105	29% (30)	47.58 ± 11.98		40.5 ± 7.8							Retrospective cohort Level III
Alqahtani et al. [30]	2019	109	9% (10)	17.6 ± 2.2		33 ± 4.7			2	6		24	Retrospective cohort Level III
Fayed et al. [32]	2019	58	41% (24)	48.2 ± 11.8		41.5 ± 8.2		3	17	9	GORD 11	12	Retrospective cohort Level III
James et al. [24]	2020	100	14% (14)	45 ± 9	106.39 ± 20.24	38.41 ± 5.44		4	29		Dyslipidaemia 13	12	Retrospective cohort Level III
Bhandari et al. [28]	2020	53	19% (10)	40.54 ± 13.79	89.12 ± 16.2	34.78 ± 5.2		10	15	6	Hypothyroidism 14 , GORD 4	12	Retrospective cohort Level III
Lopez-Nava et al. [19]	2020	11	64% (7)	42.7 ± 5.6	111.1 ± 12.3	36.9 ± 2.8						6	Prospective cohort Level III
Espinet-Coll et al. [31]	2020	88	31% (27)	46.1 ± 12.3	110.71 ± 17.9	39.4 ± 4.69		11	35	8	Arthropathy 13	12	Retrospective cohort Level III
Fiorillo et al. [33]	2020	23	30% (7)	41	115.5 ± 29.6	39.5		2	3	5	Arthropathy 7	6	Retrospective cohort Level III
Huberty et al. [34]	2021	49	6% (3)	37.6 ± 9.9	93.3 ± 8.8	34.8 ± 2.7						12	Randomised Control Trial Level I
Pizzicannella et al. [20]	2021	86	29% (25)	46.6 ± 12.8	120.9 ± 25.6	43.2 ± 8.6		27	30	48	Dyslipidaemia 11 Arthropathy 7	12	Retrospective cohort Level III
Li et al. [35]	2021	24	75% (18)	55.6 ± 9.2	157.9 ± 49.1	49.9 ± 14.4		15	29	17	GORD 3	12	Prospective cohort Level III
Jagtap et al. [25]	2021	26	38% (10)	41.5 ± 9.58	99.43 ± 21.89	36.55 ± 5.07	8.71 ± 1.55	13	19		Dyslipidaemia 17	12	Prospective cohort Level III
Asokkumar et al. [21]	2021	35	43% (15)	43.6 ± 11.3	93.2 ± 16	34 ± 4.9	8 ± 0.8	8	17		Fatty liver 14 Liver cirrhosis 3	6	Retrospective cohort Level III
Lopez-Nava et al. [48]	2021	199	29% (58)	44.6 ± 10	110 ± 19.7	39.4 ± 5.4						24	Retrospective cohort Level III
Sharaiha et al. [37]	2021	216	32% (70)	46 ± 13		39 ± 6	5.8 ± 1	67				60	Prospective cohort Level III
Sarkar et al. [50]	2022	90	39% (35)	39.7 ± 11.6		38.7		46	46	21		27.2	Retrospective cohort Level III
Alqahtani et al. [26]	2022	6036	11% (664)	34 ± 10		ESG 32.5 ± 3.1 LSG 32.9 ± 3.5		ESG 112 LSG 350	ESG 101 LSG 118		Dyslipidaemia ESG 62, LSG 163	36	Retrospective cohort Level III
Polese et al. [44]	2022	27				36 ± 9			10				Prospective cohort Level III
Abu Dayyeh et al. [45]	2022	77	12% (9)	47.3 ± 9.3	98.4 ± 12.3	35.5	5.8 ± 0.8	18	38			12	Randomised control trial Level I
Bhandari et al. [46]	2022	612	19% (188)	40.7 ± 12.6		34.3 ± 5						48	Prospective cohort Level III
Gkolfakis et al. [47]	2022	48	17% (8)	41.9 ± 9.5		34 ± 2.5							Randomised control trial Level I
Matteo et al. [12]	2022	18	44% (8)	41.2 ± 5.9		41.2 ± 5.9		4	12	6		24	Retrospective cohort Level III
Manos et al. [49]	2022	191	9% (18)	36.9		33.7						12	Retrospective cohort Level III

### Short- and Medium-Term Weight Loss

**Fig. 2:**
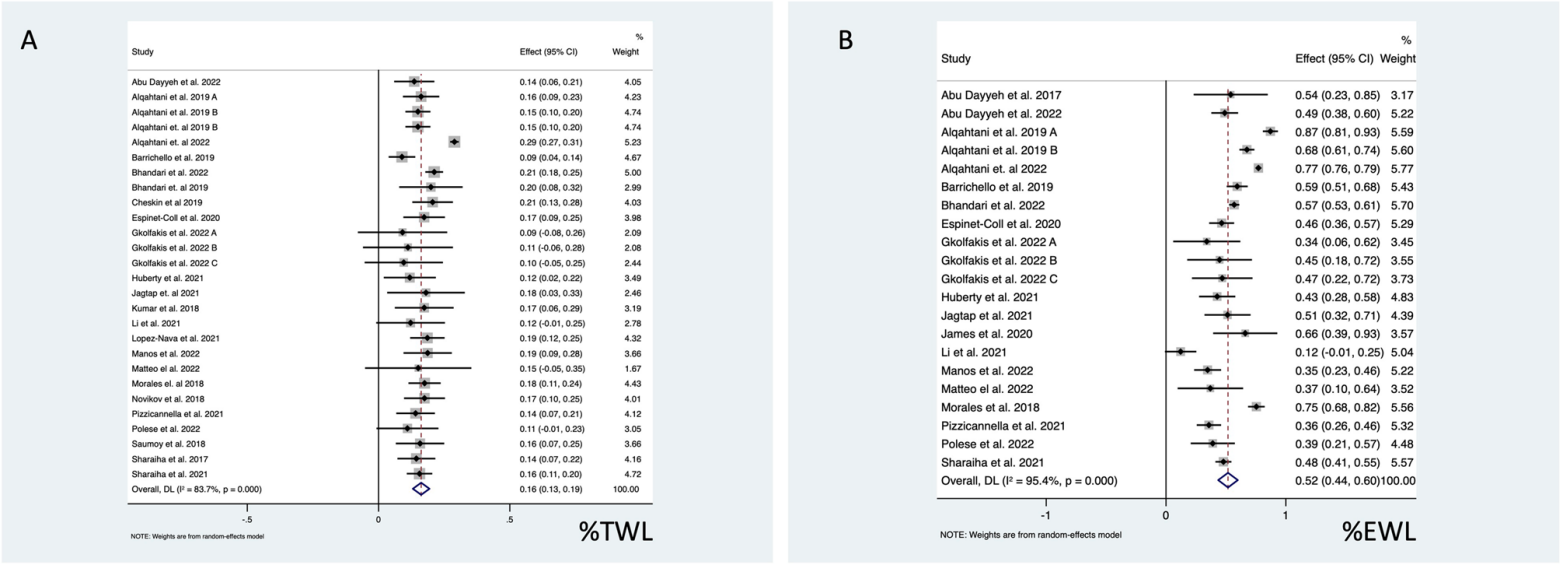
Forest plot of **A** %TWL and **B** %EWL 12 months after ESG

Short-term (1 year) %TWL data was published following 5659 ESGs and 1 year %EWL after 4852 interventions. Pooled analysis of the 23 studies recording %TWL demonstrated 16.2% (95% CI 13.1–19.4%; *I*^*2*^ = 83.7%) reduction of total body weight, and the 18 studies reporting %EWL suggested 51.7% (95% CI 43.5–59.9%; *I*^*2*^ = 95.4%) reduction in excess weight (Fig. 2).

**Fig. 3:**
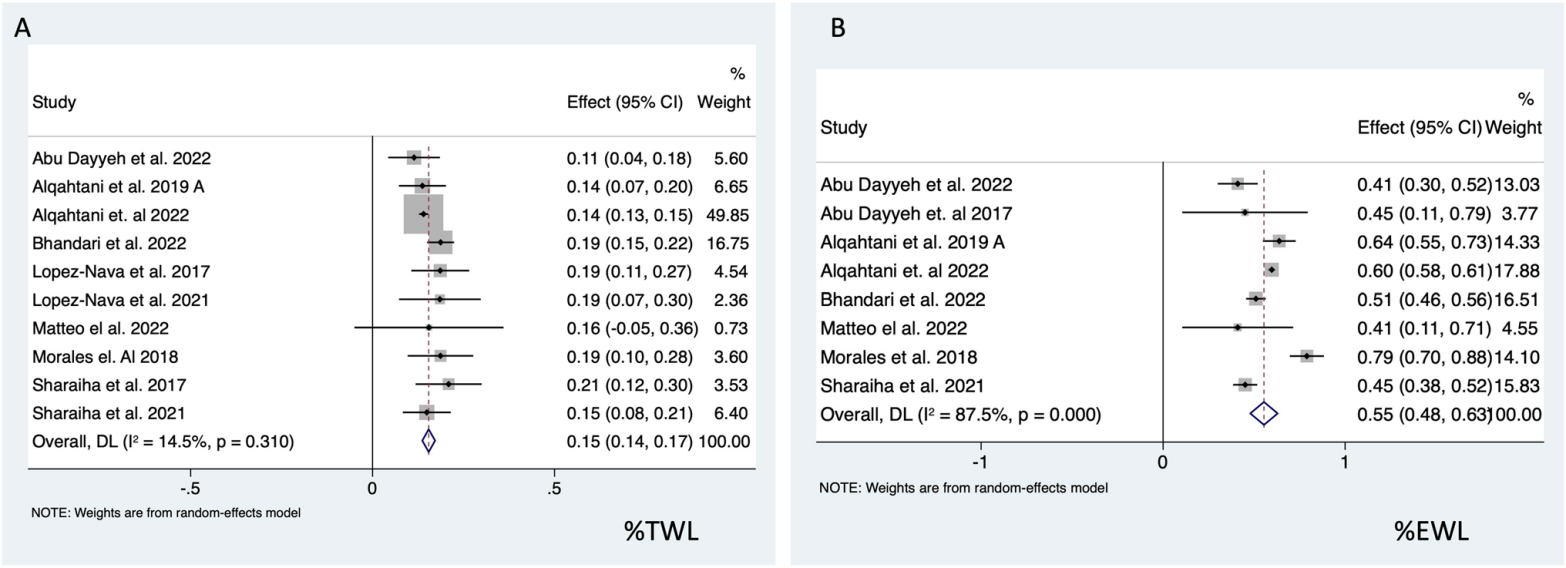
Forest plot of **A** %TWL and **B** %EWL over medium term (2–5 years) following ESG

Medium-term %TWL following ESG was recorded in ten studies investigating outcomes for 4040 patients. Random effect analysis demonstrated 15.4% (95% CI 13.7–17.2%; *I*^*2*^ = 14.5%) reduction of total body weight over the medium-term period. Percentage EWL over the medium-term was recorded in eight studies of 3837 patients and suggested a 51.8% (95% CI 47.9–62.9%; *I*^*2*^ = 87.5%) reduction of excess weight (Fig. 3.). One article described a loss of more than 50% of patients from follow-up year 3 to year 4 [46]. Hence, in our analysis, we included outcome following 3 years of follow-up but did perform the analysis with the 4 years follow-up data that did not show any difference.

### Remission of Obesity-Related Comorbidities

**Fig. 4:**
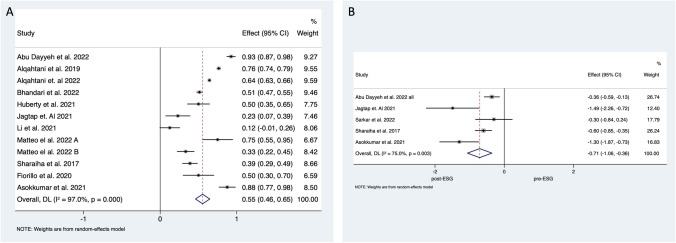
Forest plot of **A** diabetes mellitus remission and **B** reduction of HbA1c reduction 12 months after ESG

Remission of diabetes mellitus was reported in 12 studies (5034 patients including 461 diabetics) (Fig. 2). Pooled analysis demonstrated that 55.4% (95% CI 46–64%; *I*^*2*^ = 97.0%) of diabetic individuals went into remission. Four of these studies presented baseline and 12 months post-ESG follow-up HbA1c results [25, 37, 45, 50], and authors of one study provided these results separately [21]. Pooled analysis demonstrated 0.71% (95% CI − 1.06 to − 0.35%; *I*^*2*^ = 97.0%) decrease in the concentration of HbA1c which corresponds to 7.8% (95% CI 4–11%; *I*^*2*^ *= 75.0%*) improvement (Fig. 4).

**Fig. 5:**
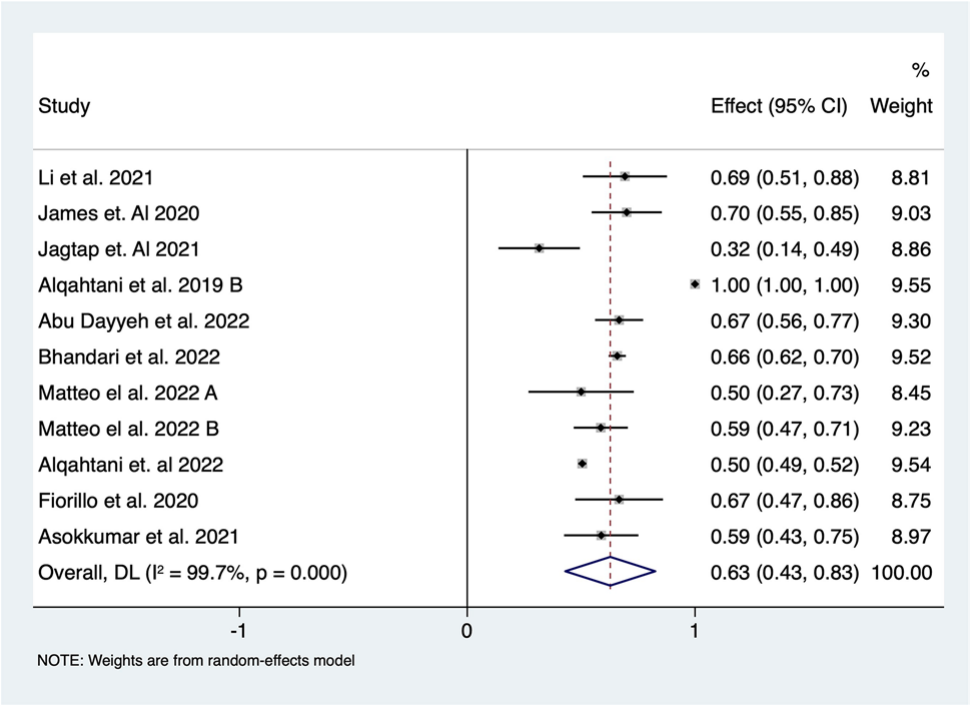
Forest plot of remission of hypertension following ESG

Hypertension outcomes were reported in 11 studies (4933 patients with 606 diagnosed with hypertension) (Fig. 3). Remission of hypertension was observed in 383 individuals corresponding to a 62.8% (95% CI 43−82%) success rate. Three studies recorded pre- and postoperative systolic blood pressure. Alongside decreasing antihypertensive medication, pooled analysis suggested a 6.8 mmHg (95% CI 9.5−4.1%; *I*^*2*^ *= 99.7%*) decrease in resting systolic blood pressure (Fig. 5).

Dyslipidaemia outcomes were reported in eight studies (4835 ESGs with 580 patients diagnosed with dyslipidaemia) (Fig. 4). Pooled data analysis demonstrated remission of dyslipidaemia in 401 patients corresponding to a 56.3% (95% CI 49−63%; *I*^*2*^ *= 93.1%*) weighted mean reduction in disease prevalence. Change in the serum level of low-density lipoprotein (LDL) was investigated in 199 patients in three studies demonstrating a 3.7 mg/dl (95% CI − 13.6–6.1%; *I*^*2*^ *= 56.9*) or 1.3% reduction (95% CI − 5–3%). One study reported an increase in the level of LDL after ESG [38]. The same three articles also published outcomes of triglyceride for the same patients before and after ESG. All of these studies demonstrated a reduction of 48.3 mg/dl (95% CI − 3.4 to − 93.1%; *I*^*2*^ *= 91.9*) in the serum level of triglyceride following ESG which corresponds to a 21.6% (95% CI 6−37%) improvement (Fig. 5).

**Fig. 6:**
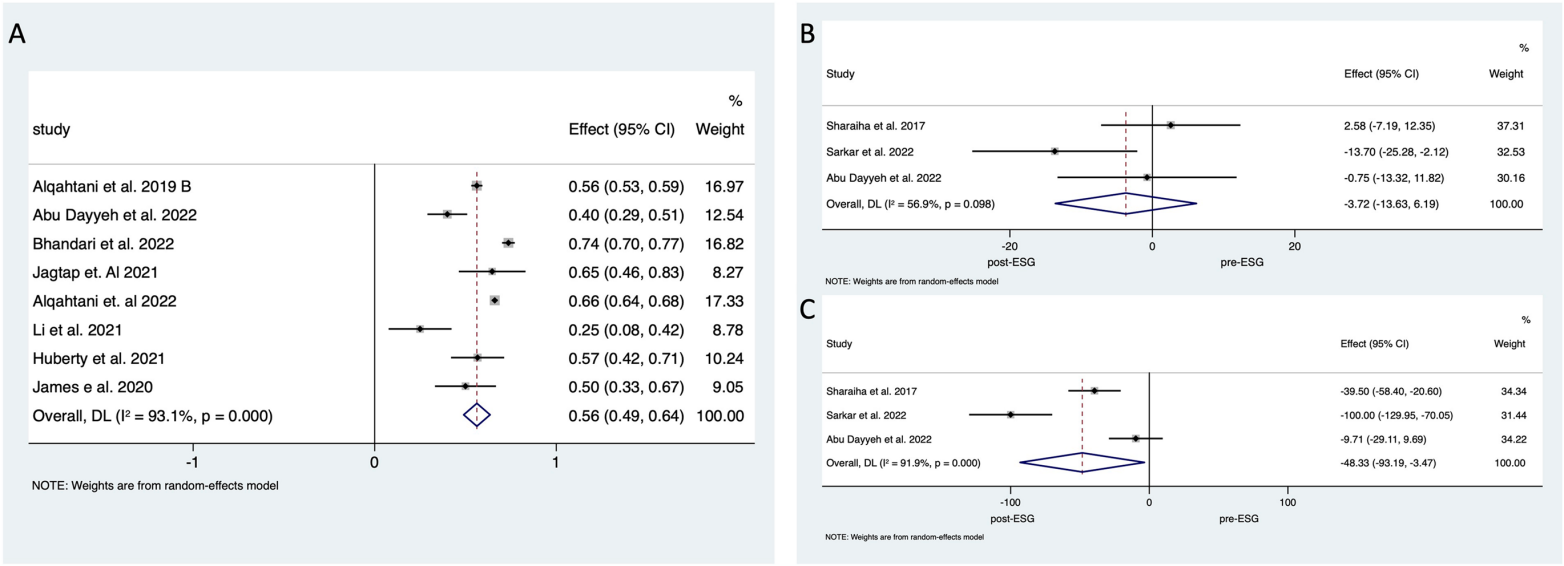
Forest plot of **A** resolution of dyslipidaemia, **B** change in triglyceride, and **C** low-density lipoprotein following ESG

**Fig. 7:**
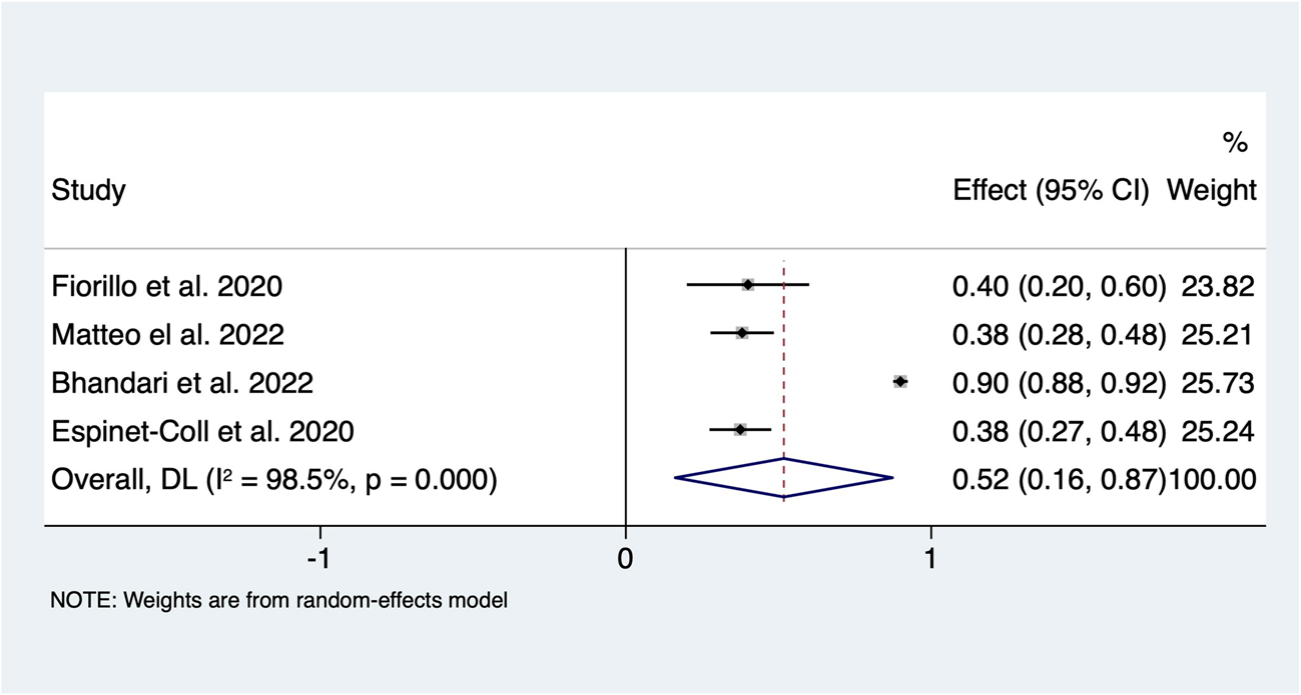
Forest plot of resolution of obstructive sleep apnoea following ESG

Pre-ESG obstructive sleep apnoea was recorded in 480 patients from 4 articles investigating 807 ESGs (Fig. 6). Overall, 51.7% (95% CI 16.2–87.3%; *I*^*2*^ *= 98.5*) of patients went into remission following intervention. No studies directly investigated liver disease, but there were four studies (*n* = 235 patients) which recorded alanine transaminase (ALT) and aspartate aminotransferase (AST) before and after ESG. A reduction of 18.2 IU (95% CI 8–28%) in ALT level and 14.6 IU (95% CI 1.9–27.2%) in AST level were observed following intervention. This corresponds to a 36.8% (95% CI 19.7–54%) improvement in ALT level and 27.9% improvement in AST level following ESG (Fig. 7.).

In terms of medium-term durability of ESG, data was extracted from all articles publishing results for at least 2 years. Out of the ten studies, five did not present the frequency of revision. In the other five articles, there were 148 out of the 4032 patients that required revision including 96 patients who underwent re-do endoscopic intervention and 52 patients who were converted to LSG. Indication for revision included insufficient weight loss, weight regain and abdominal pain. The overall weighted mean for revision was 3.67% (95% CI 3.61–3.69%).

## Discussion

This systematic review and meta-analysis reports weight loss outcomes and obesity-associated comorbidity resolution following ESG up to 5 years. The ultimate aim of all bariatric interventions is to achieve sustainable and clinically relevant weight loss. The pooled analysis of data from 35 studies containing data from 7525 patients confirmed that ESG can lead to sustained weight loss in the medium-term which is associated with significant improvements in obesity-associated comorbidities.

Although surgery is the most effective treatment for morbid obesity [51], a significant proportion of patients may not wish to undergo and/or be unsuitable for conventional surgery (e.g. due to multiple previous abdominal operations). Therefore, a non-surgical weight loss procedure with a favourable risk profile and durable weight loss addresses the needs of this patient group. By reducing the size of the gastric reservoir, ESG delays gastric emptying and induces early satiety which are thought to be the primary mechanisms for the sustained weight loss identified in this meta-analysis [8]. A large multicentre RCT (‘MERIT’) comparing ESG with lifestyle intervention has now shown that ESG can provide 45.1% EWL at 12 months compared with lifestyle modification alone, demonstrating the superiority of ESG over conservative treatment [52]. The accumulated data in this meta-analysis from multiple independent centres performing this procedure confirms that ESG is effective at inducing both obesity-associated comorbidity resolution and clinically significant weight loss.

Although there were no RCTs directly comparing ESG with LSG, resection of the fundus during a LSG removes the major source of ghrelin, a key orexigenic hormone, whereas ESG is a fundus-sparing procedure. ESG might therefore be expected to induce less weight loss than LSG. Whereas LSG can achieve a mean TWL of 23.7% after 5 years [53], our analysis found an average TWL of 15.4% after ESG in the medium-term. Data from comparative non-randomised studies comparing ESG with LSG, including a propensity-score matched analysis of 3018 patient pairs [26], suggested a mean difference in TWL of ~10% between LSG and ESG in the medium-term. It has also been proposed that weight loss and metabolic enhancement may be greater in patients with type 1 diabetes mellitus who are undergoing definitive bariatric surgery due to the immediate BRAVE (bile flow alteration, reduction of gastric size, anatomical gut rearrangement and altered flow of nutrients, vagal manipulation and enteric hormonal modulation) effects of surgery. These features, which include ESG intervention, can result in cascade effects on gut microbiome and local metabolism (intestinal gluconeogenesis and adipokine fluxes) enhancing weight loss [54]. Studies understanding the mechanism of ESG mapped on to the BRAVE effects and its downstream effectors should be considered.

Although the results of this study suggest that weight loss induced by ESG is less than LSG (pending robust clinical comparison trials), ESG has the added advantages of being an endoluminal, safe and organ-preserving procedure. These characteristics are likely to appeal to patients with obesity who would otherwise be reluctant to undergo conventional bariatric surgery. In addition, the average length of stay following ESG is an overnight stay which translates to reduced service costs [20]. Although the equipment can carry a higher cost than some surgical equipment on account of being in its early commercial cycle, the shorter recovery time and few reported adverse events support ESG in becoming a cost-effective solution. In terms of the durability of the procedure, our results demonstrated similar frequency for revision as described following LSG [55].

However, there is considerable heterogeneity in the outcomes that have been reported, which may be due to the heterogeneity of eligibility criteria and patient demographics amongst the mainly nonrandomised observational studies. Another important factor may be the lack of robust quality assurance of the procedures being performed which is becoming an essential component of intervention-based trials [52]. Saumoy et al. showed that efficacy of ESG was obtained after a minimal procedural volume of 38 ESGs, confirming that procedural quality is directly linked to outcome [39]. The MERIT RCT did include standardised training and proctoring of endoscopists, but did not include performance monitoring to ensure procedural quality was maintained throughout the trial. Future RCTs should include methods to ensure procedural quality to reduce variation in clinical outcome and improve the reliability of study findings.

This data supports the role of ESG as an option for patients who choose not to undergo and/or are unsuitable for conventional surgery, but there may be additional indications for ESG. Although the mean BMI of included studies was 37.7 kg/m^2^, one study evaluated ESG in high-risk patients (BMI > 50 kg/m^2^, unfit and/or impenetrable abdomen) and found that ESG could induce EWL of 29.1% at 12 months in this patient group [35]. ESG may also be an option as a safe revisional procedure after LSG in the setting of sleeve dilatation [56]. This is particularly important given the chronic nature of obesity and the likelihood of revisional procedures being required after LSG, particularly those performed in younger patients. Thus, the safety and organ-sparing nature of ESG makes it attractive as an initial revisional procedure after LSG. Further studies are warranted to explore the role of ESG for these indications.

The multidisciplinary team must have an active role in patient selection and follow-up to support weight loss following ESG, and the approach should be tailored to each individual patient to optimise outcomes. The decision to proceed with ESG should be guided by preoperative assessment, including medical and weight history, physical examination, laboratory tests, nutritional and psychological or psychiatric counselling. Lopez-Nava et al. [57] demonstrated that a high compliance with the follow-up post-ESG is associated with higher weight loss at one year. Furthermore, ESG is expected to have implications on endoscopy and surgery training programmes. It is a procedure that requires experience and training in endoscopic suturing in order to effectively and safely place full-thickness sutures in the stomach. Specialist bariatric gastroenterologists and surgeons will require training courses in endoscopic suturing, such as in ex vivo porcine specimens or virtual reality, at an early stage as recommended by the American Society for Gastrointestinal Endoscopy [58, 59]. Strengths of this review include the large number of included studies and the length of follow-up of up to 5 years. Limitations include the high heterogeneity of data, unavoidable duplication of data, difficult standardisation of the data limited quality assurance and risk of bias as the majority of studies were non-randomised unblinded cohort studies without sham control arms. This review has not directly analysed the risk of long-term weight recurrence after ESG after initial weight loss, short and long-term complications (e.g. acid reflux), need for revision of ESG (e.g. re-tightening or surgery) or cost-effectiveness of ESG.

In conclusion, through a meta-analysis of outcomes that have been reproduced in multiple independent centres, this review has demonstrated that ESG can generate sustained weight loss in the short- and medium-term and resolution of obesity-associated comorbidities for patients with moderate obesity. Future larger and higher quality studies, including results of ongoing RCTs [60], are needed to evaluate the role of ESG compared to other procedures and for additional indications, such as for patients with super obesity, high-risk patients and as a revisional procedure.
